# A Case Study of Emphysematous Cystitis in a Non-diabetic Patient Following Empagliflozin Use: An Uncommon Presentation

**DOI:** 10.7759/cureus.61150

**Published:** 2024-05-27

**Authors:** Nicolette Busuttil, Rami Khalaf, Ian Galea, Jonathan Calleja

**Affiliations:** 1 Surgery, Mater Dei Hospital, Msida, MLT; 2 Medicine, Queen Mary University of London, Gozo, MLT; 3 Family and Community Medicine, Gozo General Hospital, Gozo, MLT; 4 Geriatrics, Gozo General Hospital, Gozo, MLT

**Keywords:** cystitis, emphysema cystitis, sodium-glucose cotransporter-2 (sglt-2) inhibitor, complicated urinary tract infection, heart failure with reduced ejection fraction, empagliflozin, emphysematous cystitis

## Abstract

Emphysematous cystitis (EC), a rare urinary tract infection characterized by gas accumulation in the bladder walls, is predominantly seen in diabetic patients. This case study discusses an 83-year-old non-diabetic male with end-stage heart failure who presented with symptoms of EC after being administered empagliflozin. The unique presentation suggests a possible link between empagliflozin and EC in non-diabetic heart failure patients, a connection previously undocumented in medical literature, emphasizing the importance of vigilant monitoring and further research in this area.

## Introduction

Emphysematous cystitis (EC) presents a challenging and potentially life-threatening urinary tract infection characterized by the accumulation of luminal gas within the bladder walls and surrounding tissues [1, 2]. Although the precise pathophysiology of this condition remains poorly understood, it is typically attributed to facultative anaerobic microorganisms [1, 3]. While EC is most frequently observed in diabetic individuals, it is important to recognize that non-diabetic patients can also be affected, often due to an array of underlying risk factors, to be discussed further below [1, 2, 4].

In the realm of medical literature, there are still no reported cases of emphysematous cystitis associated with the use of empagliflozin in non-diabetic patients suffering from heart failure with reduced ejection fraction. This unique and unprecedented scenario underscores the significance of our case study, which aims to shed light on this uncharted territory and provide valuable insights into the management and understanding of emphysematous cystitis in non-diabetic individuals with distinct clinical backgrounds.

## Case presentation

Patient presentation

An 83-year-old male residing at an elderly home presented with gross hematuria. The patient had a severe congestive heart failure exacerbation two weeks prior to the episode of hematuria, which was treated with intravenous furosemide, strict fluid input, and output monitoring via a catheter, daily weight monitoring, and daily renal function assessment. An echocardiogram was performed due to the patient's deteriorating condition, revealing severe left ventricular systolic dysfunction. The cardiologist recommended that the patient would benefit from further anti-failure medication in accordance with guideline-directed medical therapy. The furosemide dose was adjusted and eventually switched to oral bumetanide. Spironolactone was increased to 20 milligrams (mg) and empagliflozin 10 mg was started. The patient lost 10.5 kilograms (kg) of fluid and improved after two weeks. The patient remained stable from the congestive heart failure point of view at that time. The chief complaint was gross hematuria with the presence of blood clots, dysuria, and increased urinary frequency. The patient denied experiencing chills, rigors, difficulty passing urine, or abdominal pain, which are typically associated with systemic infections or abdominal pathology.

Past medical history

The patient had a history of multiple comorbidities, including congestive heart failure (CHF), atrial fibrillation (AF), and a vertebral fracture, which was conservatively treated with a Kendall brace. The patient's cognitive abilities were intact, demonstrated by a mini-mental state examination score of 26 out of 30. The primary reason for the patient's admission to the elderly home was due to social issues. The patient used a walker for support but maintained independence in feeding. 

An echocardiogram result showed as follows: the heart was in atrial fibrillation (AF), and the heart rate was recorded between 110 to 115 beats per minute. The examination showed that the left ventricle (LV) was borderline dilated, exhibiting global hypokinesia and a significantly impaired LV systolic function. Even though the visual assessment of the left ventricular ejection fraction (LVEF) suggested it was less than 20%, measurements based on the modified Biplane Simpson's assessment revealed an LVEF of 26%. The aortic valve was identified as a trileaflet structure with mild aortic regurgitation and no evident aortic stenosis. The echocardiogram also highlighted mild to moderate mitral regurgitation. The right ventricle appeared to be of normal size and function, with no detected tricuspid regurgitation. There was a noticeable severe biatrial dilation. Additionally, the aortic root dimensions were found to be normal, and there wasn't any pericardial effusion. The inferior vena cava (IVC) was also dilated. To conclude, the patient was determined to have severe LV systolic dysfunction.

The patient was taking rivaroxaban 20 mg daily (for AF), spironolactone 25 mg daily (for CHF), omeprazole 20 mg daily, folic acid one tablet daily, carvedilol 6.25 mg twice daily, bumetanide 2 mg in the morning and 1 mg in the evening, and empagliflozin 10 mg daily.

Physical exam

The patient appeared to be neither distressed nor clinically unwell. He had a blood pressure of 96/68, a pulse rate of 110-115 bpm, a respiratory rate of 14, a blood glucose level of 6.4, and an SpO2 of 98%. Upon examination, his chest exhibited diffuse fine end-inspiratory crackles bilaterally yet had good air entry on both sides. The cardiovascular system examination revealed normal first and second heart sounds. His abdomen was soft and non-tender, with no palpable bladder.

The urine collected in the bottle displayed gross hematuria.

Diagnosis and management

The patient's vital signs were closely monitored every four hours, and several blood tests were conducted, including a full blood count, renal function test, C-reactive protein (CRP) measurement, and a group and save. The hemoglobin (Hb) level never dropped below 12.7. Initial laboratory results showed an elevated C-reactive protein (CRP) level, which rose from 71 to 129 mg/L, indicating an ongoing inflammatory process. Furthermore, the white blood cell (WBC) count was 10.37, an elevation that further suggested the potential presence of an infection.

The urinalysis revealed a pH of 6.5, urine WBC count of 500 uL, positive nitrites, proteinuria of 500 mg/dL, normal glucose and ketones, and erythrocytes count of 250 uL. 

The initial management involved monitoring the vital signs and the hematuria. Rivaroxaban was discontinued for a total of three days, and oral amoxicillin-clavulanate was initiated.

A computed tomography scan of the kidneys, ureters, and urinary bladder (CT-KUB) without contrast substance was performed to rule out any potential urological pathologies. The non-contrast scan of these areas was acquired with a slice thickness of 2.5 millimeters.

The CT-KUB scan revealed no significant consolidation. Pleural effusion was observed, measuring eighty millimeters in thickness on the right and fifty millimeters on the left.

**Figure 1:**
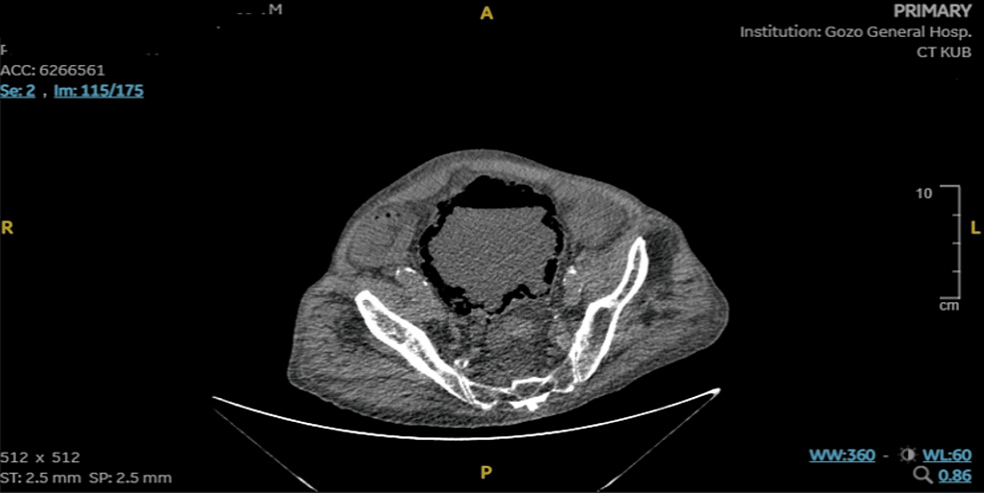
CT-KUB exhibiting the urinary bladder significantly distended with gas within its wall CT-KUB - computed tomography scan of the kidneys, ureters, and urinary bladder

Both kidneys displayed atrophic changes with small, simple cysts consistent with chronic kidney disease. Bilateral first-grade hydronephrosis was documented. Both ureters appeared normal. The urinary bladder was significantly distended, measuring 110 by 115 by 213 millimeters, and exhibited gas within its wall, as seen in Figure 1. These findings suggest emphysematous cystitis against a backdrop of chronic outflow obstruction. While it was a non-contrast scan, the liver, gallbladder, pancreas, both adrenal glands, and spleen appeared normal. Similarly, considering it was a non-colonographic study, the small and large bowels exhibited a standard appearance. A small amount of ascites was also recorded. The scan identified no aggressive lytic or sclerotic bone lesions. However, it did document a fracture of the superior endplate of the L4 vertebra, which had already been noted in a previous scan. 

In view of the CT-KUB findings, a consultation was set up with the on-call urologist, who recommended initiating piperacillin/tazobactam 4.5g, administered intravenously every six hours. A three-way catheter was inserted and used for irrigation. Subsequently, the CRP level began to decrease over three days, reaching 32 mg/L, while the WBC count dropped to 5.4mg/L.

Upon review, the urologist advised that given the patient's comorbidities, his stable clinical condition, the clearing up of the urine, and the improvement seen with intravenous antibiotics, the plan should include two weeks of intravenous antibiotics and changing the catheter to an 18 Fr silicone catheter, a trial without the catheter in four weeks, and discontinuation of empagliflozin indefinitely. An alpha-blocker was not suggested since he suffered from persistent hypotension.

The patient's urinary tract symptoms showed improvement. However, this progress was overshadowed by a subsequent exacerbation of his congestive heart failure, leading to the patient's death 20 days after this episode.

## Discussion

Pathophysiology of EC

EC is a severe urinary tract infection distinguished by the production of luminal gas and carbon dioxide. This phenomenon occurs when facultative anaerobic microorganisms undergo metabolic processes like fermentation and anaerobic respiration [1]. The literature predominantly identifies *Escherichia coli (E. coli)* as the principal causative agent, with *Klebsiella pneumoniae* also being commonly implicated [2]. Other possible causative microorganisms, such as *Proteus mirabilis*, *Clostridium perfringens*, and *Pseudomonas aeruginosa*, among others, have also been associated with EC [3]. Epidemiologically, EC tends to affect middle-aged females more frequently, with a female-to-male incidence ratio of 2:1 [4]. While diabetes stands out as the predominant risk factor, several other conditions, including neurogenic bladder, immunosuppression, chronic use of urinary catheters, persistent urinary tract infections, advanced age, and urinary stasis, contribute to the risk profile of EC [5]. Intriguingly, while more rare, EC can manifest in non-diabetic patients [6].

The pathophysiology of EC is yet to be fully understood. In diabetic patients, glycosuria, which signifies the presence of excess glucose in the urine, often provides the ideal bladder environment for gas-producing microorganisms to thrive. This is because the elevated glucose in the urine serves as an energy source for these microbes, enabling them to produce gas [7].

For non-diabetic individuals, the development of EC is hypothesized to be multifactorial. Factors like compromised immunity, diminished tissue perfusion, elevated tissue glucose concentrations, and the presence of alternative metabolic substrates like albumin and lactulose might converge to create conditions conducive for anaerobic metabolism and fermentation in the urinary bladder [8]. As medical research continues, a deeper understanding of EC's pathophysiology will pave the way for better diagnostic and therapeutic approaches.

Empagliflozin and potential relation to EC

Empagliflozin, a drug initially developed for the treatment of type 2 diabetes mellitus (T2DM), has gained traction in the medical community due to its potential efficacy in managing heart failure. Its cardiovascular benefits, as showcased in trials involving T2DM patients, spurred further research into its use in heart failure, even among non-diabetic individuals. The landmark EMPA-REG OUTCOME trial showcased empagliflozin's capability to notably diminish hospitalizations for heart failure and instances of cardiovascular death, regardless of initial heart failure status [9]. Another significant trial, EMPEROR-Reduced, exhibited empagliflozin's benefits for patients with heart failure with reduced ejection fraction (HFrEF), irrespective of their diabetes status, pointing towards a decreased risk of cardiovascular death or heart failure-related hospitalizations compared to a placebo [10].

Delving into the mechanisms underlying these outcomes, empagliflozin's hemodynamic effects stand out, primarily through osmotic diuresis that cuts down blood volume and preload, ultimately reducing cardiac strain [11]. SGLT2 inhibitors' potential in controlling myocardial fibrosis and unfavorable cardiac remodeling, as well as moderating blood pressure, further easing the cardiac workload [12].

Empagliflozin's primary mode of action revolves around the inhibition of SGLT2, a protein predominantly expressed in the proximal renal tubules. This protein plays a pivotal role in reabsorbing nearly 90% of the glucose that has been filtered back into the bloodstream [13]. By targeting and inhibiting SGLT2, empagliflozin curtails this reabsorption process, leading to heightened urinary glucose excretion, commonly referred to as glycosuria. This process subsequently reduces blood glucose concentrations, offering therapeutic benefits for T2DM patients [14].

One of the consequences of empagliflozin's mechanism, which encourages glycosuria, is the surge in glucose concentrations within the bladder. This amplified glucose presence can inadvertently offer a nurturing environment for infections, serving as a nutritional reservoir for harmful microorganisms. Consequently, this is the underlying reason for the heightened risk of urinary tract infections (UTIs) in patients administered with SGLT2 inhibitors [15].

Hazique et al. suggested a potential relationship between the use of SGLT2 inhibitors and a heightened risk of urinary tract infections. However, definitive evidence supporting this correlation was not established. The case involved a type 2 diabetic patient who experienced a urinary infection while on empagliflozin and eventually showed clinical improvement on cessation of the drug [16]. Notably, the patient's condition improved after the drug was discontinued. In a 2022 study, Uitrakul et al. analyzed the risk associated with urinary tract infections among diabetic patients undergoing various treatments, including SGLT2 inhibitors and other diabetes medications [17]. The findings indicated a more pronounced risk of urinary tract infections among those on SGLT2 inhibitors. Though this information is predominantly centered on diabetic patients, it also emphasizes the possible association of SGLT2 inhibitors with an elevated risk of emphysematous cystitis, potentially mirroring the mechanism observed in standard urinary tract infections in individuals taking the medication [18].

On the other hand, it is important to note that other studies, such as Mahling et al. (2020), examined the association between SGLT2 inhibitors and the risk of urosepsis. The meta-analysis included data from multiple randomized controlled trials and found that SGLT2 inhibitors did not significantly increase the risk of urosepsis or pyelonephritis compared to placebo. Specifically, the results showed no substantial difference in the incidence of these severe infections, suggesting that the use of SGLT2 inhibitors is relatively safe in this regard. However, the study also highlighted that while the risk of severe infections like urosepsis was not elevated, SGLT2 inhibitors are associated with a higher incidence of genital and urinary tract infections. This underscores the importance of monitoring and managing patients on these medications to mitigate the risk of these less severe but more common infections. [19]

Despite the patient not being diabetic, his current treatment regimen and diagnosis of heart failure, especially in the context of frailty and advanced age, create conditions conducive to the potential development of EC. The combination of glycosuria due to the use of SGLT2 inhibitors could have set the stage for the mentioned facultative organisms to colonize the bladder. This colonization could lead to fermentation and anaerobic respiration, producing carbon dioxide (CO2) and other gases, which then accumulate in the bladder, culminating in the observed disease manifestation [20].

Diagnosis and management of EC

The symptomatology of emphysematous cystitis varies and may not correlate with the severity of the clinical presentation [1]. The most frequently reported symptom is abdominal pain. However, patients might also be asymptomatic or present with symptoms such as pneumaturia, bothersome voiding issues, or more severe presentations like acute abdomen and urosepsis [2, 3]. In some instances, EC can be diagnosed incidentally. Diagnostic methods include ultrasound imaging, X-rays (radiography), CT scans, biopsy, and cystoscopy. Ultrasound imaging typically reveals luminal gas, which can vary based on the patient's movement or positioning. Nevertheless, CT scanning remains the gold standard diagnostic technique [4, 6, 7].

Currently, specific blood markers play no role in the assessment and management of emphysematous cystitis. However, fundamental investigations, such as urinalysis and cultures, are crucial for identifying the causative microorganism, enabling the development of personalized management plans [6].

Prompt investigation and treatment are vital to prevent severe complications, including sepsis, emphysematous pyelonephritis, bladder rupture, and death. Initial and commonly successful interventions include the administration of intravenous broad-spectrum antibiotics, insertion of a Foley catheter for bladder drainage, and strict glycemic control [7, 8]. If a large number of blood clots are present in the bladder, drainage becomes especially important to prevent tamponade of the bladder [6]. Once the causative organism is identified, narrow-spectrum antibiotics may be introduced [1]. Although rare, approximately 10% of patients may require surgical intervention, which can include debridement, partial cystectomy, total cystectomy, or even nephrectomy in the presence of emphysematous pyelonephritis. The use of hyperbaric oxygen has also been documented in literature [21].

## Conclusions

In conclusion, an unconventional case of a non-diabetic patient with a history of heart failure who developed emphysematous cystitis while on empagliflozin therapy was presented. The etiology, potential risk factors, diagnostic techniques, and prospective management strategies were examined. Additionally, the potential influence of the patient's comorbidities, along with the mechanism of action of the SGLT2 inhibitor, was postulated as the primary cause. Nevertheless, further research is essential to ascertain a correlation between these factors.
